# Interpersonal Callousness and Co-Occurring Anxiety: Developmental Validity of an Adolescent Taxonomy

**DOI:** 10.1037/abn0000235

**Published:** 2016-12-15

**Authors:** Alan J. Meehan, Barbara Maughan, Charlotte A. M. Cecil, Edward D. Barker

**Affiliations:** 1 Department of Psychology, Institute of Psychiatry, Psychology & Neuroscience, King’s College London; 2 MRC Social, Genetic and Developmental Psychiatry Centre, Institute of Psychiatry, Psychology & Neuroscience, King’s College London; 3 Department of Psychology, Institute of Psychiatry, Psychology & Neuroscience, King’s College London

**Keywords:** interpersonal callousness, anxiety, psychopathology, risk factors, Avon Longitudinal Study of Parents and Children

## Abstract

Growing evidence suggests heterogeneity within interpersonal-callous (IC) youth based on co-occurring anxiety. The developmental validity of this proposed taxonomy remains unclear however, as most previous research is cross-sectional and/or limited to adolescence. We aimed to identify low-anxiety (IC/ANX−) and high-anxiety (IC/ANX+) IC variants, and compare these groups on (a) early risk exposures, (b) psychiatric symptoms from midchildhood to early adolescence, and (c) school-based functioning. Using the Avon Longitudinal Study of Parents and Children (ALSPAC), a prospective epidemiological birth cohort, model-based cluster analysis was performed on children with complete age-13 IC and anxiety scores (*n* = 6,791). Analysis of variance was used to compare resulting clusters on (a) prenatal and postnatal family adversity and maternal psychopathology, and harsh parenting; (b) developmental differences in attention-deficit/hyperactivity disorder (ADHD), conduct disorder (CD), oppositional defiant disorder (ODD), emotional difficulties, and low pro-social behavior at 7, 10, and 13 years; and (c) teacher-reported discipline problems, along with standardized test performance. We identified a 4-cluster solution: “typical,” “low,” “IC/ANX−”, and “IC/ANX+.” IC/ANX+ youth showed the highest prenatal and postnatal levels of family adversity and maternal psychopathology, highest levels of ADHD, CD, ODD, and emotional difficulties, greatest discipline problems, and lowest national test scores (all *p* < .001). IC/ANX+ also showed a distinct pattern of increasing psychopathology from age 7 to 13 years. Adolescent IC subtypes were successfully validated in ALSPAC across multiple raters using prenatal and early postnatal risk, repeated measures of psychopathology, and school-based outcomes. Greater prenatal environmental risk among IC/ANX+ youth suggests an important target for early intervention.

## General Scientific Summary

Children who display interpersonal callousness (IC; i.e., deceitful behavior, lack of empathy/remorse) have historically been thought to show reduced anxiety or fear. This study, however, identified a subgroup of youth who show IC alongside elevated anxiety. Compared to a low-anxiety IC group, these high-anxiety IC children experienced greater prenatal and postnatal adversity, presented higher levels of co-occurring psychiatric disorders, had greater behavioral problems at school, and performed worse in national standardized school tests.

Youth who display a callous interpersonal style (e.g., superficial charm, deceitful behavior, lack of empathy/remorse, shallow affect) appear to be at a higher risk for more severe, aggressive, stable, and resistant patterns of conduct problems (Byrd, Loeber, & Pardini, 2012; Frick, Ray, Thornton, & Kahn, 2014). Indeed, *DSM–5* includes a specifier for callousness (termed “limited prosocial emotions”) in the diagnostic criteria for conduct disorder (CD; American Psychiatric Association, 2013). This interpersonal callousness (IC) corresponds to Factor 1 of the two-factor model of psychopathy (Harpur, Hare, & Hakstian, 1989), which has subsequently been divided into separate “interpersonal” and “affective” components in three-factor (Cooke & Michie, 2001) and four-factor models (Hare & Neumann, 2006). However, among youth, it is increasingly recognized that co-occurring anxiety denotes further heterogeneity within this construct. Specifically, a distinction has been made in relation to adult psychopathy (Karpman, 1941, 1948; Skeem, Johansson, Andershed, Kerr, & Louden, 2007), and subsequently in adolescence (Kahn et al., 2013; Vaughn, Edens, Howard, & Smith, 2009), between low-anxiety (i.e., IC/ANX−) and high-anxiety (IC/ANX+) variants. These may show etiological variation: IC/ANX– is conceptualized as having a strong heritable basis, whereas IC/ANX+ youth are thought to become both callous and anxious in response to social adversity, for example, parental maltreatment or bullying by other children (Barker & Salekin, 2012; Humayun, Kahn, Frick, & Viding, 2014; Kimonis, Skeem, Cauffman, & Dmitrieva, 2011).

Comparable adolescent studies, mainly referring to callous-unemotional (CU) traits, suggest that compared to low-anxiety groups, high-anxiety CU youth have higher comorbidity, such as greater externalizing and internalizing behavior (Euler et al., 2015; Kahn et al., 2013), conduct problems (Fanti, Demetriou, & Kimonis, 2013), delinquency (Vaughn et al., 2009), aggression, and violence (Docherty, Boxer, Huesmann, O’Brien, & Bushman, 2016; Kimonis et al., 2011). This group also experience greater adversity, as measured by past histories of abuse or trauma (Kimonis, Fanti, Isoma, & Donoghue, 2013; Kimonis, Frick, Cauffman, Goldweber, & Skeem, 2012; Kimonis et al., 2011; Sharf, Kimonis, & Howard, 2014; Tatar, Cauffman, Kimonis, & Skeem, 2012; Vaughn et al., 2009). Prenatal maternal risk, including anxiety and depression, has been linked to increased CU at age 13 (Barker, Oliver, Viding, Salekin, & Maughan, 2011), but no study to date has compared high- and low-anxiety variants on this association. Similarly, although a systematic review concluded that negative parenting dimensions (e.g., negative discipline, harsh parenting) consistently predicted higher callousness (Waller, Gardner, & Hyde, 2013), a recent study that distinguished ANX− and ANX+ variants found no difference in parental negativity or harsh discipline between the two groups (Humayun et al., 2014). Finally, although some studies have included teacher reports of behavior problems in the school context (e.g., Docherty et al., 2016), academic performance has not been compared between IC/ANX− and IC/ANX+ variants.

Although growing evidence supports the validity of IC variants, a number of limitations have been identified in the existing literature. First, with some recent exceptions (Cecil et al., 2014; Fanti et al., 2013; Humayun et al., 2014), this line of research has generally employed cross-sectional designs. Consequently, the extent to which the above comorbidities might be developmentally linked with IC, and whether early environmental exposures confer differential risk for future maladjustment between IC variants, remains unclear. Second, much of this research has focused on forensic, or otherwise institutionalized, populations. Relatively little work has investigated the degree to which findings generalize to nonclinical, community-based samples. Third, the literature primarily centers on adolescence, with little examination of early childhood risk factors; to date, the earliest evidence of differences between these variants is at age 7 (Humayun et al., 2014). Fourth, few studies assess sex differences, having typically relied on exclusively male or female samples. One recent study reported a greater prevalence of girls in its equivalent IC/ANX+ variant, whereas more boys were found in the IC/ANX– variant (Euler et al., 2015). However, little has been done to investigate whether these variants associate differently with psychiatric comorbidities or risk factors depending on sex.

The present study sought to overcome these common limitations by examining the developmental validity of IC variants using a prospective, epidemiological, and mixed-gender birth cohort. Consistent with previous adolescent research, we hypothesized that IC/ANX− and IC/ANX+ variants would be identifiable at age 13, and would be differentiated based on (a) prenatal and postnatal risk exposures, including harsh parenting; (b) preadolescent symptoms of psychopathology; and (c) discipline problems and academic performance in the school environment. Given the longitudinal nature of the data, we also sought to examine whether (d) variants differed on the developmental course of psychopathology from midchildhood (age 7) through to early adolescence (age 13). Finally, we took potential differences between boys and girls into account by examining main and interaction effects for sex throughout analysis.

## Method

### Sample

The Avon Longitudinal Study of Parents and Children (ALSPAC) is an ongoing epidemiological study established to understand how genetic and environmental characteristics influence health and development in parents and children. All pregnant women resident in the former Avon Health Authority of southwest England with expected delivery dates between April 1, 1991 and December 31, 1992 were eligible for recruitment. This resulted in a cohort of 14,541 pregnancies, of which 13,988 singletons/twins were alive at 12 months of age. ALSPAC is broadly representative of the UK population as a whole compared to 1991 National Census Data (Boyd et al., 2013). It should be noted that the ethnic composition of the initial sample, although consistent with the Avon area at the time of recruitment, was primarily White (96.09%). Ethical approval was obtained from the ALSPAC Law and Ethics Committee, as well as various Local Research Committees. Please note that the study website contains details of all available data, through a fully searchable data dictionary: http://www.bris.ac.uk/alspac/researchers/data-access/data-dictionary/.

### Measures

#### Clustering measures

##### Interpersonal callousness

A six-item questionnaire was completed by mothers when their child was 13 years old (Moran, Ford, Butler, & Goodman, 2008). Items were rated on a three-point scale, from *not true* to *certainly true*: (i) makes a good impression at first, but people tend to see through them after getting know them; (ii) shallow or fast-changing emotions; (iii) usually genuinely sorry if they have hurt someone or acted badly (reverse coded); (iv) can seem cold-blooded or callous; (v) keeps promises (reverse coded); and (vi) genuine in their expression of emotions (reverse coded). Items were selected on the basis of factor analyses of scales measuring CU traits (Frick, Bodin, & Barry, 2000; Frick, O’Brien, Wootton, & McBurnett, 1994). The measure correlated highly (*r* = .81) with the CU scale of the Antisocial Process Screening Device in 182 children aged 9–17 displaying antisocial behavior (Moran et al., 2009). Internal consistency was good (α = .75). Due to the small number of items, the measure was maintained as a single scale in analysis.

##### Anxiety

We used a measure of the likelihood of “any anxiety disorder” at age 13; this encompassed separation anxiety disorder, generalized anxiety disorder, specific phobia, social phobia, agoraphobia, and/or panic disorder. This anxiety score was generated from the Development and Well Being Assessment (DAWBA), originally developed for the British Child Mental Health Surveys (R. Goodman, Ford, Richards, Gatward, & Meltzer, 2000). Using parent-reported symptoms, preliminary *DSM–IV* psychiatric diagnoses were generated using a well-defined computerized algorithm (see http://www.dawba.com), producing six-level ordered-categorical “probability bands” for each disorder, ranging from <0.1% to >70% probability of diagnosis. These “bands” have functioned well as ordered-categorical measures when evaluated in two large-scale national samples, showing dose-response associations with mental health service contacts, and similar associations with potential risk factors as clinician-rated diagnoses (A. Goodman, Heiervang, Collishaw, & Goodman, 2011).

#### Early risk exposure

##### Family adversity

Measures of environmental risk were collated under the Family Adversity Index (Bowen, Heron, Waylen, Wolke, & the ALSPAC Study Team 2005), assessed during pregnancy at 18–32 weeks gestation, and postnatally between 0 and 2 and 2–4 years. This index measures 17 family based risk factors across eight risk domains: age of mother; housing adequacy; no educational qualifications; financial difficulties; poor partner relationships; maternal substance abuse; and maternal criminal behavior. An item was rated 1 if adversity was present, with scores summed to create a scale. We created two cumulative adversity scores: one prenatal and one postnatal.

##### Maternal psychopathology

Anxiety and depression in mothers were assessed by the Crown-Crisp Experiential Index (CCEI; Crown & Crisp, 1979) and the Edinburgh Postnatal Depression Scale (EPDS; Cox, Holden, & Sagovsky, 1987), respectively. Assessments were conducted at 18 and 32 weeks prenatally, and postnatally at 8 weeks, 8 months, 21 months, and 33 months. The anxiety subscale of the CCEI comprises eight self-reported items, measuring the frequency with which anxiety-related feelings and behaviors are experienced along a four-point scale (*never* to *very often*). The EPDS is a 10-item self-report questionnaire of depressive symptoms experienced in the last seven days. Latent variables combining depression and anxiety into overall “maternal psychopathology” were created for the prenatal and postnatal periods, with higher values suggesting greater psychopathology.

##### Harsh parenting

Disciplinary parenting practices were assessed by two items each at ages 2 and 4, asking the mother, “When you are at home with your child, how often do you do the following”: (i) shout at him/her; and (ii) slap him/her. The original response scale (1 = *every day* to 5 = *rarely/never*) was reversed so that higher scores reflected harsher parenting. Resulting scores from both ages were combined into a single latent factor.

#### Childhood psychopathology

##### Externalizing DSM–IV disorder diagnoses

At ages 7, 10, and 13 years, measures of externalizing behavior were drawn from parental ratings on the DAWBA. As with anxiety at age 13 (see above), computer-generated, clinician-reviewed “probability bands” derived from these ratings ranged from <0.1% to >70% probability of *DSM–IV* psychiatric diagnosis. Specifically, we examined diagnoses of attention-deficit/hyperactivity disorder (ADHD; including hyperactive, inattentive, and combined subtypes), conduct disorder (CD), and oppositional defiant disorder (ODD).

##### Emotional difficulties and low pro-social behavior

Also at ages 7, 10, and 13 years, emotional difficulties and pro-social behavior were measured using mother reports on subscales of the Strengths and Difficulties Questionnaire (SDQ), which has previously shown good reliability and validity (R. Goodman, 1997). Subscales comprised five items each, rated on three-point scales (*not true*, *somewhat true*, and *certainly true*). To measure low pro-social behavior, pro-social SDQ items (“considerate of other people’s feelings,” “shares readily with other children,” “helpful if someone is hurt,” “kind to younger children,” “volunteers to help others”) were reverse coded, such that higher scores reflected the disregard for others and lack of empathy that form a key component of IC. Some of these items have previously been employed in assessment of childhood callousness, although it should be noted that commonly recognized components of broader IC are not included in this construct (Viding, Blair, Moffitt, & Plomin, 2005; Whelan, Stringaris, Maughan, & Barker, 2013).

#### School functioning

##### Child discipline problems

The teacher version of the SDQ (R. Goodman, 1997) was completed when the child was in the third year of compulsory education (i.e., Year 3; age 7–8). The five-item conduct problems subscale was used to capture teacher ratings of the child’s disruptive behavior in the classroom. Also at age 7, during DAWBA data collection, teachers reported on the degree to which they complained about the child’s overactivity, poor concentration, and impulsiveness within the previous six months. Possible responses were *not at all*, *a little*, or *a lot*, with a higher summary “teacher complaints” score indicating a higher frequency of complaints.

##### Child academic performance

National standardized test data were used to evaluate academic progress throughout primary education. Year-on-year progress of UK children is divided into “key stages,” with compulsory national tests at the end of each stage. For Key Stage 1, at the end of Year 2 (i.e., 6–7 years of age), English (reading, writing) and Mathematics are examined. For Key Stage 2, at the end of Year 6 (i.e., 10–11 years), tests of English, Science, and Mathematics are administered. Key Stage I and 2 scores were created by summing the national curriculum level scores (Levels 1–8) achieved for each subject.

### Attrition and Missing Data

Participants with complete IC and anxiety data at age 13 were selected for analysis, resulting in a sample of 6,791 (49.99% boys). Using multivariate logistic regression, we tested the extent to which study variables predicted exclusion from the analytic sample. Odds ratios (ORs) showed that mothers excluded from the present analysis were more likely to experience postnatal family adversity (*OR* = 1.05, 95% CIs [1.02, 1.09]). However, mothers *included* in analysis were more likely to experience greater adversity (*OR* = 1.33, 95% CIs [1.25, 1.41]) and anxiety/depression during pregnancy (*OR* = 1.07, 95% CIs [1.01, 1.14]), with their children more likely to show conduct disorder symptoms at age 7 (*OR* = 1.22, 95% CIs [1.06, 1.40]). On all other study variables, included and excluded participants did not differ.

### Statistical Analyses

Analyses were performed using SPSS Statistics version 22, Mplus version 7.11 (Muthén & Muthén, 2012), and the *mclust* package in R version 3.2.1 (Fraley, Raftery, Murphy, & Scrucca, 2012). Given the relatively large sample size (and high statistical power), we applied stringent significance thresholds throughout analyses; specifically, *p* < .001 for main effects and *p* < .01 for interactions. In reporting results, we first present significant three-way interactions, followed by two-way interactions, and finally main effects. Given the hierarchical nature of interaction terms, we refrained from discussing significant lower-ordered terms in the presence of significant higher-ordered terms; that is, we did not discuss two-way interactions that were nested in significant three-way interactions, and did not discuss main effects that were nested in significant two-way interactions. Effect sizes were interpreted using Cohen’s (1988) suggested guidelines. Cohen’s *d* (small = 0.2; medium = 0.5; large = 0.8) is reported for differences between two groups, and (partial) eta squared (η^2^; small = 0.01; medium = 0.06; large = 0.14) is given for ANOVA main and interaction effects. Analysis comprised three steps:

#### Step 1: Cluster identification

Consistent with past adolescent studies (Docherty et al., 2016; Kimonis et al., 2012; Kimonis et al., 2011; Tatar et al., 2012), we performed model-based cluster (MBC) analysis on IC and anxiety scores at age 13. Though not uncommon in this field (see Docherty et al., 2016; Euler et al., 2015), our cluster analysis included only two variables: one for IC and one for anxiety. A data-driven approach, MBC tests the relative fit of 10 models, which vary in their assumptions about the distribution of clusters (spherical, diagonal, or ellipsoidal), and whether clusters have equal or variable size, shape, and orientation in space (Skeem et al., 2007). Within each of these models, the number of clusters is varied from one to nine; thus, 90 different cluster solutions are examined. Goodness-of-fit is determined by the Bayesian Information Criterion (BIC). Generally, the model with the lowest BIC value is preferred. Further conventions around BIC values in MBC are discussed elsewhere (see Raftery, 1995).

#### Step 2: Early risk exposure

We compared mean differences between resulting IC/anxiety groups on prenatal and early postnatal measures of family adversity, maternal psychopathology, and harsh parenting, using a multivariate analysis of variance (MANOVA) where all five risk exposures were entered simultaneously. We also investigated potential interactions based on sex.

#### Step 3: Psychopathology

Separate mixed ANOVAs (i.e., within-subjects [ages 7, 10, 13] and between-subjects [cluster, sex]) were conducted for each measure of psychopathology, in order to examine group and/or sex differences while also accounting for developmental change over time.

#### Step 4: School functioning

Finally, teacher ratings of the child’s classroom behavior, along with standardized test scores, were compared between clusters and sexes using univariate ANOVAs, taking potential Cluster × Sex interactions into account.

## Results

### Step 1: Cluster Identification

The best-fitting MBC model was a four-cluster solution, which was diagonal in distribution and had variable volume and shape (BIC = −46,635.48). This offered a better fit than the second-best (BIC = −46,642.45) and third-best-fitting models (BIC = −46,743.62). The BIC difference of 6.97 between the best and second-best solution constituted “strong” support for the better-fitting model, representing odds of at least 20:1 that it provided a better fit (Raftery, 1995). The average classification certainty, or posterior probability that an individual was correctly assigned to a cluster, was 75.4%; a value >70% is suggested as indicating clear classification (Nagin, 2005).

**Figure 1 fig1:**
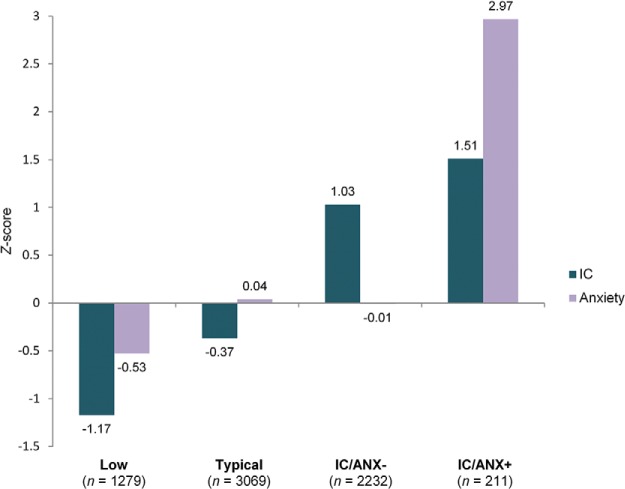
*Z*-score mean profiles of interpersonal callousness (IC) and anxiety at age 13 for the four-cluster solution. See the online article for the color version of this figure.

Overall main effects were significant for both IC, *F*(3, 6787) = 6,452.36, *p* < .001, η^2^ = .74 (95% CIs [.73, .75]), and anxiety, *F*(3, 6787) = 1,106.96, *p* < .001, η^2^ = .33 (95% CIs [31, .34]). Tukey’s honestly significant difference was used for pairwise comparisons. To aid comparison, mean *z-*scores for each cluster group on IC and anxiety are presented in Figure 1. The largest cluster (*n* = 3,069, 51.3% female) presented average scores for IC and anxiety that most closely resembled the overall group means, and was labeled “typical.” The second cluster (*n* = 1,279, 47% female) had the lowest IC and anxiety scores in the sample; thus, we labeled it “low.” The third cluster (*n* = 2,232, 49.3% female) showed significantly higher IC than the “typical” group; however, the two clusters did not significantly differ on anxiety (*p* = .301). Consequently, we labeled it “IC/ANX−.” Finally, the fourth cluster (*n* = 211, 57.8% female), the smallest overall, was significantly higher on both IC and anxiety than IC/ANX−, and was labeled “IC/ANX+.” A significant difference on IC between IC/ANX− and IC/ANX+ groups was somewhat unexpected, given our hypothesis that these IC subtypes would be chiefly discriminated by anxiety. However, further exploration using Cohen’s *d* effect sizes revealed that the difference between IC/ANX+ and IC/ANX−, though small-to-medium (*d* = 0.44), was substantially smaller in magnitude than observed effect size differences between the other clusters (average *d* = 2.77).[Fig-anchor fig1]

### Step 2: Early Risk Exposure

Table 1 presents descriptive statistics for each cluster, and the sample as a whole, on prenatal and postnatal risk exposures, along with MANOVA results. Overall, there were significant main effects for cluster membership across all five risk domains (all *p* < .001). Effect sizes were small-to-medium (η_*p*_^2^ = .02–.05). Post hoc comparisons applying Bonferroni corrections revealed that, with only one exception, the IC/ANX+ cluster had the highest levels of risk exposure. Specifically, IC/ANX+ scores were significantly higher than IC/ANX− for all environmental exposures except harsh parenting, where these clusters did not differ. Effect size differences between IC/ANX+ and IC/ANX– were similarly small-to-medium (*d* = .33–.45; see supplemental Table S1, available online). The IC/ANX– cluster in turn scored higher than the typical cluster across all risk factors. Finally, average risk scores in the typical cluster were significantly higher than the low cluster in all comparisons, with the exception of prenatal family adversity, where group differences were nonsignificant. We found no Cluster × Sex interactions for any of these comparisons.[Table-anchor tbl1]

### Step 3: Psychopathology

Table 2 presents means and standard deviations on dimensional psychopathology scores at age 7, 10, and 13 years for each cluster. Given expected mean differences between boys and girls, these are reported separately by sex (total descriptive statistics for each cluster are presented in online supplemental Table S2). From initial inspection, we observed a consistent pattern of increasing levels of psychopathology across clusters, level differences between boys and girls, and evidence of differential developmental change from age 7 to 13.[Table-anchor tbl2]

**Figure 2 fig2:**
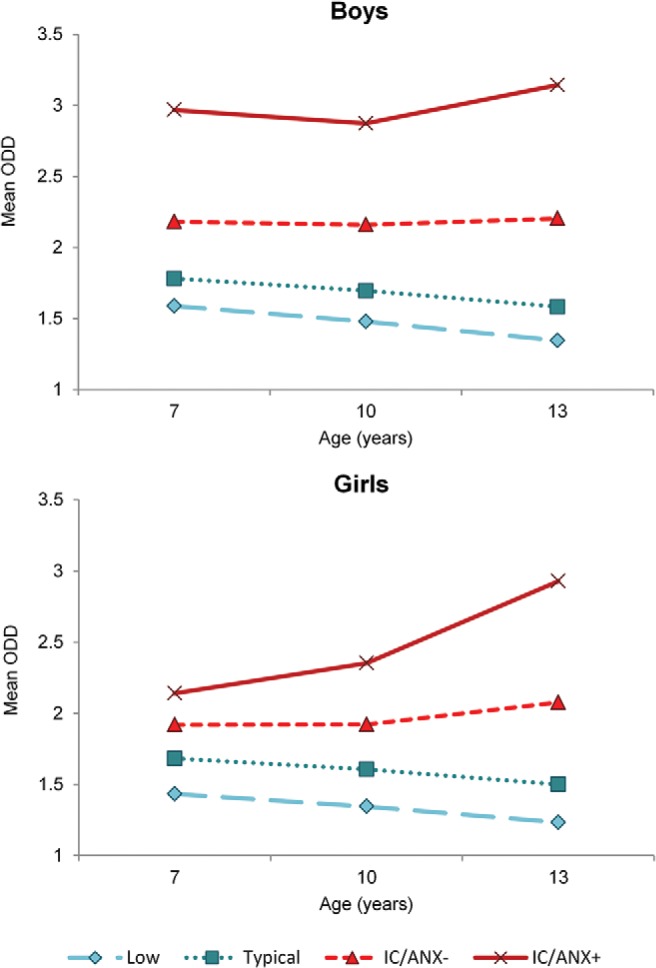
Age × Cluster × Sex interaction for oppositional defiant disorder (ODD). See the online article for the color version of this figure.

Mixed ANOVAs of 3 (age 7, 10, and 13) × 4 (cluster) × 2 (sex) design were used to test these differences more formally. Greenhouse-Geisser corrections were used where the sphericity assumption was violated. First, ODD showed a significant Age × Cluster × Sex interaction (*p* < .01). Mean cluster scores at each age are plotted separately for boys and girls (see Figure 2). Additional nominally significant (i.e., *p* < .05) three-way interactions for CD and emotional difficulties are presented in supplemental Figure S1. For both boys and girls, mean ODD in the low and typical clusters generally decreased across age. With regard to IC/ANX–, levels were relatively stable across age for both sexes. Finally, for IC/ANX+ boys, ODD decreased slightly from age 7 to 10, before increasing thereafter, whereas the girls showed a consistent increase across age. The effect size here was small (η_*p*_^2^ = .002), which may reflect the fact that boys and girls, although differing in means, showed broadly similar developmental patterns from age 7 to 13.[Fig-anchor fig2]

**Figure 3 fig3:**
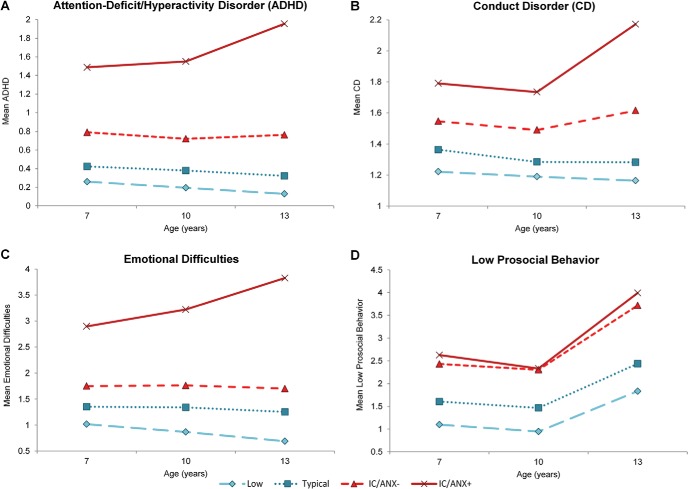
Age × Cluster interactions for attention-deficit/hyperactivity disorder (A), conduct disorder (B), emotional difficulties (C), and low pro-social behavior (D). See the online article for the color version of this figure.

Next, we found significant Age × Cluster interactions for ADHD, CD, emotional difficulties, and low pro-social behavior, presented in Figure 3. For ADHD, CD, and emotional difficulties (Figures 3A–3C), low and typical clusters showed decreases across age, IC/ANX− clusters remained relatively stable, and IC/ANX+ clusters showed consistent increases from age 7 to 13 (although CD decreased slightly from age 7 to 10 before increasing thereafter). For low pro-social behavior (Figure 3D), scores for all four clusters decreased from age 7 to 10 before increasing from age 10 to 13. CD also showed an Age × Sex interaction at *p* < .01 (see supplemental Table S2), although the resulting pattern was the same for boys and girls, who showed decreasing scores from age 7 and 10 and increasing scores between age 10 and 13.[Fig-anchor fig3]

In addition, significant Cluster × Sex interactions were noted for ADHD, CD, ODD, and low pro-social behavior, presented in supplemental Figure S2. Here, for both boys and girls, mean levels differed between all clusters in the following order: *IC/ANX+* > *IC/ANX−* > *Typical* > *Low*, with the exception of low pro-social behavior, where IC/ANX− and IC/ANX+ did not differ significantly (*p* = .83). Boys also showed higher scores than girls across all four of these comorbidities. However, in general, the mean increases between clusters for boys were more marked than for girls, as reflected in these significant interaction effects. Beyond interactions, emotional difficulties showed a significant main effect for cluster, with mean differences between clusters following the above order of effect. Overall effect size differences for the main effect of group ranged from medium to large (η_*p*_^2^ = .11–.24), whereas those for sex were smaller (η_*p*_^2^ = .002–.03). Furthermore, between IC/ANX+ and IC/ANX–, for ADHD, CD, and ODD, effect size differences (see Table S1) were small-to-medium at ages 7 (*d* = .27–.42) and 10 (*d* = .25–55), and medium-to-large at age 13 (*d* = .47–.74).

### Step 4: School Functioning

Finally, groups were compared on childhood discipline problems and academic performance. Table 3 presents descriptive statistics and ANOVA results for these variables, for both boys and girls. All main effects for group were significant at *p* < .001. Small-to-medium effect sizes noted for these main effects (η_*p*_^2^ = .01–.05) were encouraging, given that these analyses were across raters (parents vs. teachers or standardized test scores). IC/ANX+ youth had significantly higher levels of teacher-reported complaints and conduct problems, and lower average test scores at both Key Stages 1 and 2, compared to the IC/ANX− group. The magnitude of these differences was small (*d* = −32–.32). IC/ANX−, in turn, has significantly higher discipline problems and worse test performance than typical children. The typical and low groups did not differ on test performance at Key Stage 1 (*p* = .12). With regard to sex, boys scored higher on measures of discipline problems, and had lower test scores at Key Stage 1 than girls. Effect sizes for these sex differences were small-to-medium (η_*p*_^2^ = .009–.04). Finally, Cluster × Sex interactions were observed for teacher complaints and conduct problems, whereby boys showed larger mean increases between clusters compared to girls (see supplemental Figure S3).[Table-anchor tbl3]

## Discussion

In examining the developmental validity of adolescent IC/ANX– and IC/ANX+ variants, the current study is unique in three ways. First, using a prospective epidemiological birth cohort, we examined risk exposure beginning in pregnancy and extending to early childhood; as far as we are aware, these represent the earliest assessments of risk in IC subtyping research to date. Second, we examined developmental trends in co-occurring psychopathology (i.e., ADHD, CD, ODD, emotional difficulties, and low pro-social behavior) at 7, 10, and 13 years of age. Third, we validated IC variants in both home and school environments, with a novel focus on teacher-reported school-based outcomes and national test performance.

In this sample, it was IC/ANX+ youth who were the smallest and most pathological in terms of experience of early risk and co-occurring difficulties, based on mother and teacher reports. We note that our IC/ANX− group was considerably larger than what has previously been reported. This may be due to the fact that analyses were based on a relatively low-risk epidemiological sample using a dimensional IC measure, as opposed to measures of IC that have validated clinical cut-offs. Consequently, this may reflect a more normative group of IC youth, rather than indexing a highly pathological group. Nevertheless, the IC/ANX− variant still had higher levels of environmental risk exposure and co-occurring difficulties, and poorer academic outcomes, than the more typical group. The IC/ANX− group’s developmental patterns of psychopathology also appeared relatively stable, in contrast to the increasing trend of IC/ANX+ and the decreasing trends of the typical and low groups. Therefore, the presence of elevated callous interpersonal functioning may still associate with future negative outcomes for the IC/ANX− group.

Overall, our findings extend current knowledge of callous subtypes in three main ways. First, previous research consistently demonstrates that IC/ANX+ experience greater adversity than IC/ANX− youth (Kimonis et al., 2013; Sharf et al., 2014; Tatar et al., 2012). We support and extend this pattern by showing that IC/ANX+ youth experienced the highest levels of family adversity (including both socioeconomic disadvantage and interpersonal stressors) and maternal psychopathology (anxiety, depression) starting in pregnancy. These patterns were also maintained through to age 4. Given that a large body of literature finds that maternal stress during pregnancy can associate with atypical fetal development in a manner that increases offspring susceptibility for postnatal disease and maladjustment (Gluckman, Cutfield, Hofman, & Hanson, 2005), it may be that the IC/ANX+ group’s increased range of psychopathology can be partially attributed to this prenatal exposure. It is worth noting however that these IC/ANX− and IC/ANX+ groups did not differ on levels of harsh parenting. Although previous literature reports prospective associations between harsh or negative parenting and increased CU traits (Barker et al., 2011; Waller et al., 2013), the only other study to compare similar variants on early parenting likewise found no difference between the two (Humayun et al., 2014). Further research is needed to clarify the impact of parenting behavior within the IC construct itself.

Second, extant literature highlights greater comorbid psychopathology for IC/ANX+ compared to IC/ANX− (Euler et al., 2015; Fanti et al., 2013). Our study supported these findings, for both externalizing and internalizing psychopathology, and also extended them: IC/ANX+ youth were the only group to show an overall increasing developmental trend for all types of psychopathology from age 7 to age 13. This contrasted with our low and typical clusters, which showed a decreasing trend across age; this aligns with normative preadolescent developmental trajectories previously identified for externalizing and internalizing problems (Bongers, Koot, van der Ende, & Verhulst, 2004; Kim & Cicchetti, 2006). IC/ANX− youth, meanwhile, had relatively stable levels of psychopathology over time, albeit with some evidence of an increasing trend for CD. It is also worth noting that IC variants did not differ on low pro-social behavior, although both scored significantly higher than the typical and low groups. A few of the “pro-social” items used here have previously been employed in measurement of childhood callousness (Viding et al., 2005; Whelan et al., 2013), which may suggest that IC/ANX− and IC/ANX+ are similar in IC prior to age 13, that is, our first point of direct IC assessment.

Third, sex differences have rarely been examined in callous variant studies. We found a higher number of girls than boys in the IC/ANX+ cluster. This resembled the profile of sex differences observed in the only other published mixed-gender study to date (Euler et al., 2015), and reflects a greater prevalence of anxiety and depression in girls compared to boys (Rutter, Caspi, & Moffitt, 2003). Consistent with wider research on externalizing and internalizing problems (Costello, Mustillo, Erkanli, Keeler, & Angold, 2003; Crick & Zahn-Waxler, 2003), boys showed higher levels of ADHD, CD, ODD, low pro-social behavior, and discipline problems than girls, whereas girls had greater emotional difficulties and performed better on national tests compared to boys. However, for boys and girls alike, IC/ANX+ designated the highest levels of comorbid psychopathology. We had one counterintuitive finding with regard to sex: In our IC/ANX+ variant, girls showed increasing developmental trends for CD, ODD, and emotional difficulties, whereas boys decreased between age 7 and 10 before increasing from age 10 to 13. Girls, compared with boys, have previously shown increasing trajectories for internalizing symptoms in early adolescence, which we replicated via emotional difficulties (Hankin, 2009). However, for CD and ODD, results may imply that girls, who are generally less likely to develop externalizing problems, show more persistent strains of these difficulties when present at more severe levels (i.e., the IC/ANX+ variant).

### Clinical Implications

In supporting the developmental validity of IC/ANX+ and IC/ANX− variants in adolescence, the present findings offer two main clinical implications. First, given strong associations between prenatal and early postnatal environmental risk and IC/ANX+, it is possible that IC levels in these youth could be reduced if the relevant adverse social conditions were identified and targeted (Barker & Salekin, 2012). Although a previous review concluded that IC traits conferred risk for poorer treatment outcomes (Hawes, Price, & Dadds, 2014), this finding may not be as consistent once potential heterogeneity within IC is taken into account. No research to date has compared treatment outcomes between IC variants, however. We offer suggestive evidence that, for the IC/ANX+ variant, interventions very early in development, including prenatally, could be beneficial. Prenatal and early postnatal risks are implicated in risk for psychopathology across the life course (Shonkoff, Boyce, & McEwen, 2009), and are advocated elsewhere as important starting points for preventive interventions (Tremblay, 2010). Therefore, this study identifies risk factors (i.e., family adversity and maternal psychopathology) that could potentially represent core treatment targets, from the prenatal period onward, that may be responsive to early intervention.

Second, the high psychiatric comorbidity in both IC variants, from both maternal and teacher reports, offers support for the expansion of IC’s clinical utility within disorder diagnosis. At present, the “limited prosocial emotions” specifier for CD is the primary diagnostic representation of IC in *DSM–5*. However, applying Robins and Guze’s (1970) criteria for psychiatric diagnosis validation, Herpers, Rommelse, Bons, Buitelaar, and Scheepers (2012) posited that callousness may represent a “cross-disorders construct”; that is, that IC may act as a specifier for further disorders beyond CD. Our own findings, where higher scores on ADHD, CD, ODD, and emotional difficulties were observed for both IC variants compared to more typical youth, support this proposal. Moreover, the consistently higher symptom levels evidenced in IC/ANX+ youth compared to IC/ANX− suggests that anxiety could prove useful as a further subtyping characteristic for any callous specifier, in terms of differentiating risk for more severe levels of maladjustment. Consequently, IC’s potential as a cross-disorders construct should be further developed with the aim of improving clinical diagnosis and better categorizing highly callous patients.

### Strengths and Limitations

The study was characterized by a number of strengths, including its large sample size, broad scope, developmentally focused longitudinal design, multiinformant data, mixed-gender recruitment, and use of validated diagnostic bands corresponding to *DSM–IV*. However, a number of limitations must be acknowledged.

First, the six-item ALSPAC IC measure did not allow for examination of subfactors of psychopathy. Although this measure purported to measure CU traits, and has been used to represent CU in previous research (e.g., Barker et al., 2011; Cecil et al., 2014), it is best characterized as Factor 1 of the original two-factor psychopathy model (Harpur et al., 1989), denoting a manipulative interpersonal style as well as a callous affective disposition. However, Factor 1 has been further divided into “interpersonal” and “affective” facets in more recent three- and four-factor models. Previous studies have used separate measures of “interpersonal” and “affective” components when examining similar anxiety-based subgroups. However, results have been mixed. Skeem et al. (2007) found their high-anxiety psychopathic group had lower “interpersonal” and “affective” scores than a low-anxiety psychopathic group. Kimonis et al. (2011) found higher “interpersonal” factor scores in their high-anxiety psychopathic group compared to the low-anxiety psychopathic group, but no difference on the “affective” facet. Other studies have found no differences between subgroups on either of the two components (Kimonis et al., 2012; Tatar et al., 2012). Future research should endeavor to better align with more recent theoretical conceptualizations of psychopathy where possible, in order to further examine potential group differences on the individual “interpersonal” and “affective” facets.

Second, only one specific measure of IC was available (at age 13), precluding analysis of stability across childhood. Although this construct has been found to be reasonably stable (Frick, Kimonis, Dandreaux, & Farell, 2003), and our use of “low pro-social” items that previously represented aspects of the IC construct provides some reassurance for stability, future work should clarify the temporal pattern observed for callous subtypes.

Third, although we utilized general anxiety symptoms to identify subtypes, research has also used specific types of anxiety (e.g., physiological anxiety, worry/oversensitivity; Kimonis et al., 2011), or even included depression and trauma symptoms (e.g., Kahn et al., 2013). Future work may want to continue to examine different conceptualizations of internalizing problems, as a recent review has proposed that psychopathic individuals may show deficits in threat responsivity and detection, rather than the subjective experience of fear or anxiety (Hoppenbrouwers, Bulten, & Brazil, 2016).

Fourth, although ALSPAC represents a broad, representative spectrum of socioeconomic backgrounds, the cohort features relatively low rates of ethnic minorities, necessitating replication with more ethnically diverse samples.

Fifth, like most large longitudinal cohorts, ALSPAC has experienced attrition over time, with children of younger and more socially disadvantaged mothers more likely to be lost in follow-up. However, we found relatively few systematic differences between excluded and included cases, with little evidence that the most severely affected children were underrepresented. Furthermore, previous studies of ALSPAC found that, although attrition affected prevalence rates of externalizing and internalizing disorders, associations between risks and outcomes remained intact, although conservative of the likely true effects (Wolke et al., 2009).

## Conclusion

The current findings supported the developmental validity of distinct low-anxiety (IC/ANX−) and high-anxiety (IC/ANX+) IC variants using a longitudinal, community-based sample. We found differences between IC variants on environmental risk exposures as early as pregnancy, extending this taxonomy to an earlier time-point than previous research. Furthermore, IC/ANX− and IC/ANX+ youth in our sample presented significantly higher levels of ADHD, CD, ODD, emotional difficulties, and low pro-social behavior compared to more typical youth. In addition, distinct developmental trends in co-occurring psychopathology from midchildhood (age 7) to early adolescence (age 13) were noted for these variants, with IC/ANX+ youth in particular showing consistent increases across age. This taxonomy was valid across multiple raters and environments (i.e., home and school), based on differences in school functioning, and for males and females alike, although some sex differences were identified regarding relative levels of co-occurring difficulties. We highlight prenatal and early postnatal adversity as important treatment targets for IC, particularly where anxiety co-occurs, as in IC/ANX+. We also suggest that IC offers clinical utility not only as a specifier for more severe CD, but also higher levels of ADHD, ODD, and internalizing difficulties (i.e., anxiety and depression).

## Supplementary Material

10.1037/abn0000235.supp

## Figures and Tables

**Table 1 tbl1:** Means and Standard Deviations for Derived Clusters on Risk Exposures, With MANOVA Results

		*M* (*SD*)				MANOVA		
Variable	Total Sample (*n* = 6123)	(*a*) Low (*n* = 1173)	(*b*) Typical (*n* = 2771)	(*c)* IC/ANX−(*n* = 1991)	(*d*) IC/ANX+ (*n* = 188)	*F*(3, 6115)	η_*p*_^2^	*Post hoc*
Prenatal								
Family Adversity	.81 (1.22)	.63 (.97)	.76 (1.16)	.93 (1.32)	1.54 (1.93)	39.55***	.02	*d* > *c* > *b*, *a*
Maternal Psychopathology	−.11 (.90)	−.39 (.81)	−.14 (.87)	.04 (.91)	.49 (1.09)	86.47***	.04	*d* > *c* > *b* > *a*
Postnatal (birth–age 4)								
Family Adversity	1.76 (2.11)	1.39 (1.72)	1.67 (2.02)	1.97 (2.28)	3.11 (2.99)	46.83***	.02	*d* > *c* > *b* > *a*
Maternal Psychopathology	−.07 (.77)	−.34 (.67)	−.09 (.75)	.07 (.78)	.52 (1.02)	108.49***	.05	*d* > *c* > *b* > *a*
Harsh Parenting	−.01 (1.08)	−.33 (1.10)	−.05 (1.07)	.19 (1.02)	.26 (1.12)	67.45***	.03	*d, c* > *b* > *a*
*Note*. η_*p*_^2^ = partial eta squared. Descriptive statistics for the total sample are included for comparative purposes.								
*** *p* < .001.								

**Table 2 tbl2:** Descriptive Statistics for Boys and Girls in Each Cluster on Psychopathology at Ages 7, 10, and 13 Years, With Mixed ANOVA Results

	*M* (*SD*)								*F* (with η_*p*_^2^ effect size)				
	Low		Typical		IC/ANX−		IC/ANX+		Within-Subjects		Between-Subjects		
Variable	Boys (*n* = 536–557)	Girls (*n* = 482–491)	Boys (*n* = 1,173–1,199)	Girls (*n* = 1,217–1,241)	Boys (*n* = 873–890)	Girls (*n* = 842–860)	Boys (*n* = 62–64)	Girls (*n* = 85–95)	Age × Cluster × Sex	Age × Cluster	Cluster × Sex	Cluster	Sex
ADHD													
Age 7	.35 (.70)	.17 (.48)	.53 (.87)	.32 (.65)	.96 (1.15)	.62 (.92)	2.03 (1.67)	.94 (1.18)					
Age 10	.24 (.61)	.14 (.49)	.47 (.81)	.29 (.65)	.91 (1.13)	.54 (.82)	2.08 (1.56)	1.02 (1.19)	1.63	17.51***	20.75***	311.36***	169.47***
Age 13	.20 (.55)	.06 (.29)	.43 (.76)	.22 (.55)	.95 (1.12)	.58 (.85)	2.36 (1.62)	1.55 (1.44)		η_*p*_^2^ = .01	η_*p*_^2^ = .01	η_*p*_^2^ = .15	η_*p*_^2^ = .03
CD													
Age 7	1.26 (.45)	1.19 (.39)	1.36 (.51)	1.37 (.49)	1.59 (.64)	1.51 (.59)	2.03 (.94)	1.55 (.70)					
Age 10	1.22 (.42)	1.16 (.39)	1.30 (.47)	1.27 (.46)	1.52 (.60)	1.46 (.55)	1.89 (1.05)	1.58 (.66)	2.57*	23.43***	6.67***	318.04***	37.77***
Age 13	1.18 (.39)	1.14 (.35)	1.30 (.47)	1.27 (.45)	1.65 (.71)	1.58 (.66)	2.21 (1.17)	2.13 (1.12)	η_*p*_^2^ = .001	η_*p*_^2^ = .01	η_*p*_^2^ = .004	η_*p*_^2^ = .16	η_*p*_^2^ = .01
ODD													
Age 7	1.59 (.58)	1.43 (.52)	1.78 (.67)	1.68 (.59)	2.18 (.87)	1.92 (.68)	2.95 (1.35)	2.14 (.92)					
Age 10	1.48 (.57)	1.34 (.50)	1.69 (.66)	1.61 (.58)	2.16 (.87)	1.92 (.69)	2.86 (1.41)	2.35 (1.02)	4.34***	45.17***	9.97***	546.91***	88.49***
Age 13	1.35 (.55)	1.23 (.45)	1.58 (.64)	1.50 (.59)	2.20 (.91)	2.08 (.79)	3.14 (1.44)	2.93 (1.38)	η_*p*_^2^ = .002	η_*p*_^2^ = .03	η_*p*_^2^ = .006	η_*p*_^2^ = .24	η_*p*_^2^ = .02
Emo Diff													
Age 7	.97 (1.25)	1.08 (1.24)	1.26 (1.50)	1.48 (1.60)	1.65 (1.71)	1.82 (1.75)	3.05 (2.29)	2.92 (2.12)					
Age 10	.82 (1.18)	.90 (1.37)	1.20 (1.59)	1.51 (1.70)	1.65 (1.70)	1.84 (1.79)	3.06 (2.59)	3.48 (2.32)	2.55*	11.59***	1.31	201.91***	9.00**
Age 13	.61 (1.03)	.76 (1.13)	1.07 (1.42)	1.43 (1.58)	1.50 (1.68)	1.90 (1.83)	3.87 (2.57)	3.78 (2.39)	η_*p*_^2^ = .002	η_*p*_^2^ = .01		η_*p*_^2^ = .11	η_*p*_^2^ = .002
Low Pro													
Age 7	1.35 (1.53)	.86 (1.26)	1.91 (1.72)	1.33 (1.48)	2.82 (1.81)	2.02 (1.65)	3.23 (2.23)	2.31 (1.87)					
Age 10	1.16 (1.30)	.73 (1.08)	1.73 (1.54)	1.21 (1.36)	2.65 (1.79)	1.90 (1.59)	2.94 (2.25)	1.93 (1.63)	.59	22.99***	5.19**	361.62***	98.73***
Age 13	1.99 (1.23)	1.68 (1.00)	2.68 (1.57)	2.19 (1.32)	4.04 (1.92)	3.39 (1.78)	4.21 (2.27)	3.77 (1.89)		η_*p*_^2^ = .01	η_*p*_^2^ = .003	η_*p*_^2^ = .18	η_*p*_^2^ = .02
*Note.* Sample sizes varied due to missing data. ADHD = attention-deficit hyperactivity disorder; CD = conduct disorder; ODD = oppositional defiant disorder; Emo Diff = emotional difficulties; Low Pro = low pro-social behavior; η_*p*_^2^ = partial eta squared. For brevity, only *F*-statistics closely related to our hypotheses are reported. Additional ANOVA findings (age main effect, Age × Sex interaction), and total cluster descriptives not separated by sex, are available online as supplemental Table S2.													
* *p* < .05. ** *p* < .01. *** *p* < .001.													

	*M* (*SD*)								*F* (η_*p*_^2^ effect size)		
	Low (*a*)		Typical (*b*)		IC/ANX− (*c*)		IC/ANX+ (*d*)				
Variable	Boys (*n* = 323–603)	Girls (*n* = 291–543)	Boys (*n* = 679–1,287)	Girls (*n* = 714–1,404)	Boys (*n* = 526–988)	Girls (*n* = 487–983)	Boys (*n* = 33–71)	Girls (*n* = 55–103)	Cluster	Sex	Cluster × Sex
Discipline Problems											
Teacher Complaints (age 7)	.29 (.79)	.08 (.36)	.44 (.99)	.21 (.65)	.90 (1.42)	.37 (.87)	2.17 (2.06)	.46 (.93)	109.27***	248.14***	37.91***
									η_*p*_^2^ = .05	η_*p*_^2^ = .04	η_*p*_^2^ = .02
Conduct Problems (age 7–8)	.43 (.94)	.18 (.56)	.67 (1.3)	.32 (.81)	1.08 (1.71)	.44 (1.05)	1.82 (1.93)	.76 (1.14)	31.94***	62.07***	6.19***
									η_*p*_^2^ = .03	η_*p*_^2^ = .02	η_*p*_^2^ = .006
Academic Performance											
Key Stage 1 (age 6–7)	10.24 (3.38)	11.08 (3.1)	9.98 (3.44)	10.75 (3.25)	9.31 (3.52)	10.50 (3.23)	7.83 (4.39)	9.27 (3.87)	26.21***	49.79***	1.71
									η_*p*_^2^ = .01	η_*p*_^2^ = .009	
Key Stage 2 (age 10–11)	13.25 (1.69)	13.42 (1.51)	13.22 (1.73)	13.20 (1.73)	12.75 (1.89)	12.97 (1.82)	12.04 (2.33)	12.20 (2.29)	36.24***	4.3*	.66
									η_*p*_^2^ = .02	η_*p*_^2^ = .001	
*Note.* Sample sizes varied due to missing data. η_*p*_^2^ = partial eta squared. Plots of significant interactions are available in supplemental Figure S3.											
* *p* < .05. *** *p* < .001.											

**Table 3 tbl3:**
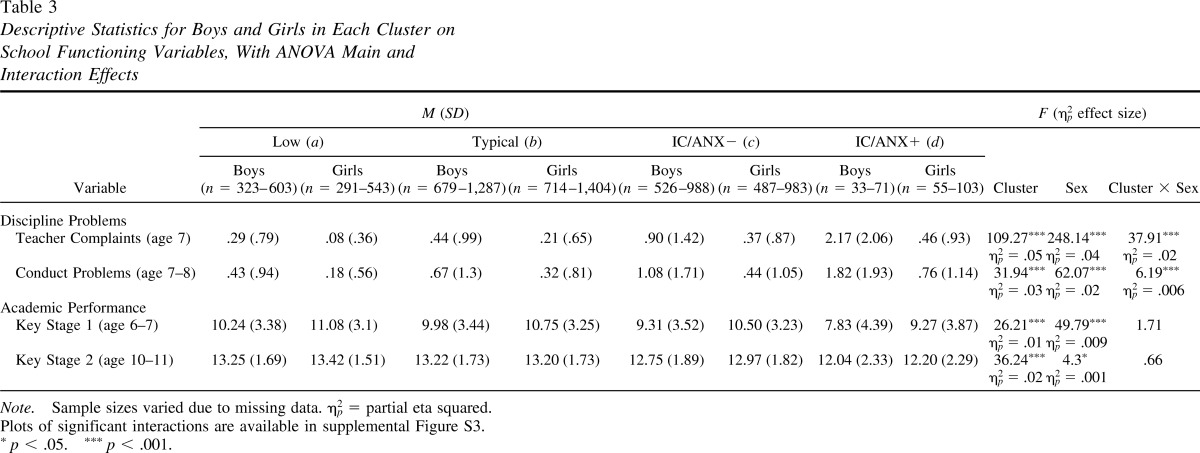
Descriptive Statistics for Boys and Girls in Each Cluster on School Functioning Variables, With ANOVA Main and Interaction Effects
