# Supplementary material for: Interpersonal Callousness and Co-Occurring Anxiety: Developmental Validity of an Adolescent Taxonomy
Source: J Abnorm Psychol. 2016 Dec 15;126(2):225–36. doi: 10.1037/abn0000235 (PMC5305415; doi:10.1037/abn0000235)
Supplement: Supplementary file 1 [file z2l006163013so1.doc]

**Supplemental Materials**

**Interpersonal Callousness and Co-Occurring Anxiety: Developmental Validity of an Adolescent Taxonomy**

**by A. J. Meehan, 2016, *Journal of Abnormal Psychology***

**http://dx.doi.org/10.1037/abn0000235**

Table S1

*Effect Sizes for Group Differences on Study Variables (Cohen’s* d*)*

|  | **Typical vs. low** | **IC/ANX- vs. typical** | **IC/ANX+ vs. typical** | **IC/ANX+ vs. IC/ANX-** |
| --- | --- | --- | --- | --- |
| **Environmental risk** |  |  |  |  |
| ***Prenatal*** |  |  |  |  |
| Family adversity | **.12^**^** | **.14^***^** | **.42^***^** | **.33^***^** |
| Maternal psychopathology | **.30^***^** | **.19^***^** | **.59^***^** | **.43^***^** |
| ***Postnatal*** |  |  |  |  |
| Family adversity | **.15^***^** | **.14^***^** | **.49^***^** | **.39^***^** |
| Maternal psychopathology | **.36^***^** | **.21^***^** | **.60^***^** | **.45^***^** |
| Harsh parenting | **.26^***^** | **.24^***^** | **.29^***^** | .06 |
|  |  |  |  |  |
| **Psychopathology** |  |  |  |  |
| ***Age 7*** |  |  |  |  |
| ADHD | **.25^***^** | **.39^***^** | **.67^***^** | **.42^***^** |
| CD | **.30^***^** | **.33^***^** | **.49^***^** | **.27^**^** |
| ODD | **.37^***^** | **.46^***^** | **.62^***^** | **.34^***^** |
| Emotional difficulties | **.24^***^** | **.22^***^** | **.76^***^** | **.60^***^** |
| Low prosocial behavior | **.34^***^** | **.47^***^** | **.52^***^** | .14 |
| ***Age 10*** |  |  |  |  |
| ADHD | **.32^***^** | **.36^***^** | **.78^***^** | **.55^***^** |
| CD | **.22^***^** | **.39^***^** | **.49^***^** | **.25^**^** |
| ODD | **.41^***^** | **.53^***^** | **.76^***^** | **.42^***^** |
| Emotional difficulties | **.35^***^** | **.22^***^** | **.82^***^** | **.66^***^** |
| Low prosocial behavior | **.37^***^** | **.51^***^** | **.46^***^** | .04 |
| ***Age 13*** |  |  |  |  |
| ADHD | **.40^***^** | **.49^***^** | **1.03^***^** | **.74^***^** |
| CD | **.32^***^** | **.54^***^** | **.77^***^** | **.47^***^** |
| ODD | **.46^***^** | **.77^***^** | **1.04^***^** | **.59^***^** |
| Emotional difficulties | **.47^***^** | **.28^***^** | **1.07^***^** | **.88^***^** |
| Low prosocial behavior | **.47^***^** | **.75^***^** | **.72^***^** | .10 |
|  |  |  |  |  |
| **School functioning** |  |  |  |  |
| Teacher complaints | **.19^***^** | **29^***^** | **.51^***^** | **.32^***^** |
| Teacher-rated conduct problems | **.21^***^** | **.21^***^** | **.43^***^** | **.26^*^** |
| Key Stage 1 Performance | **-.08^*^** | **-.14^***^** | **-.41^***^** | **-.30^***^** |
| Key Stage 2 Performance | **-.10^**^** | **-.17^***^** | **-.45^***^** | **-.32^***^** |

*Note.* ADHD = attention-deficit hyperactivity disorder; CD = conduct disorder; ODD = oppositional defiant disorder.

**^*^***p* < .05, **^**^***p* < .01, **^***^***p* < .001.

Table S2

|  | *M* (*SD*) | | | | | | |  | *F* (with**effect size)  Within-subjects | |
| --- | --- | --- | --- | --- | --- | --- | --- | --- | --- | --- |
|  | Clusters | | | | | | |  |  |  |
| Variable | Low  (*n* = 943–1031) |  | Typical  (*n* = 2,228–2,434) |  | IC/ANX-  (*n* = 1,556–1,746) |  | IC/ANX+  (*n* = 137-151) |  | Age  (7–13) | Age × Sex |
| **ADHD** |  |  |  |  |  |  |  |  |  |  |
| Age 7 | 0.26 (0.61) |  | 0.42 (0.77) |  | 0.79 (1.05) |  | 1.40 (1.50) |  | **10.27^***^**  * =* .002 | 2.36 |
| Age 10 | 0.20 (0.56) |  | 0.38 (0.74) |  | 0.72 (1.00) |  | 1.47 (1.45) |  |  |  |
| Age 13 | 0.13 (0.45) |  | 0.32 (0.67) |  | 0.77 (1.01) |  | 1.89 (1.56) |  |  |  |
| **CD** |  |  |  |  |  |  |  |  |  |  |
| Age 7 | 1.22 (0.42) |  | 1.36 (0.49) |  | 1.55 (0.61) |  | 1.75 (0.85) |  | **36.78^***^**  * =* .007 | **5.31^**^**  * =* .001 |
| Age 10 | 1.19 (0.40) |  | 1.28 (0.47) |  | 1.49 (0.57) |  | 1.71 (0.86) |  |  |  |
| Age 13 | 1.16 (0.37) |  | 1.28 (0.46) |  | 1.62 (0.69) |  | 2.17 (1.14) |  |  |  |
| **ODD** |  |  |  |  |  |  |  |  |  |  |
| Age 7 | 1.51 (0.56) |  | 1.73 (0.63) |  | 2.05 (0.79) |  | 2.49 (1.19) |  | **8.17^***^**  * =* .002 | **15.77^***^**  * =* .003 |
| Age 10 | 1.41 (0.54) |  | 1.65 (0.62) |  | 2.04 (0.79) |  | 2.57 (1.23) |  |  |  |
| Age 13 | 1.29 (0.51) |  | 1.54 (0.62) |  | 2.14 (0.86) |  | 3.02 (1.40) |  |  |  |
| **Emo Diff** |  |  |  |  |  |  |  |  |  |  |
| Age 7 | 1.01 (1.29) |  | 1.36 (1.53) |  | 1.75 (1.74) |  | 2.88 (2.19) |  | **3.716^*^**  * =* .001 | 2.79 |
| Age 10 | 0.86 (1.26) |  | 1.34 (1.64) |  | 1.76 (1.75) |  | 3.28 (2.41) |  |  |  |
| Age 13 | 0.68 (1.08) |  | 1.26 (1.52) |  | 1.70 (1.76) |  | 3.82 (2.46) |  |  |  |
| **Low Pro** |  |  |  |  |  |  |  |  |  |  |
| Age 7 | 1.12 (1.42) |  | 1.60 (1.62) |  | 2.44 (1.79) |  | 2.54 (2.09) |  | **546.87^***^**  * =* .1 | **3.96^*^**  *=* .001 |
| Age 10 | 0.96 (1.22) |  | 1.46 (1.47) |  | 2.31 (1.74) |  | 2.24 (1.91) |  |  |  |
| Age 13 | 1.85 (1.14) |  | 2.43 (1.47) |  | 3.72 (1.88) |  | 3.95 (2.06) |  |  |  |

*Total Cluster Descriptive Statistics for Psychopathology (Ages 7, 10 and 13 Years), and Additional Mixed ANOVA Within-Subject Effects*

*Note.* Sample sizes varied owing to missing data. ADHD = attention-deficit/hyperactivity disorder; CD = conduct disorder; ODD = oppositional defiant disorder; Emo Diff = emotional difficulties; Low Pro = low prosocial behavior; **= partial eta squared.

**^*^***p* < .05, **^**^***p* < .01, **^***^***p* < .001.


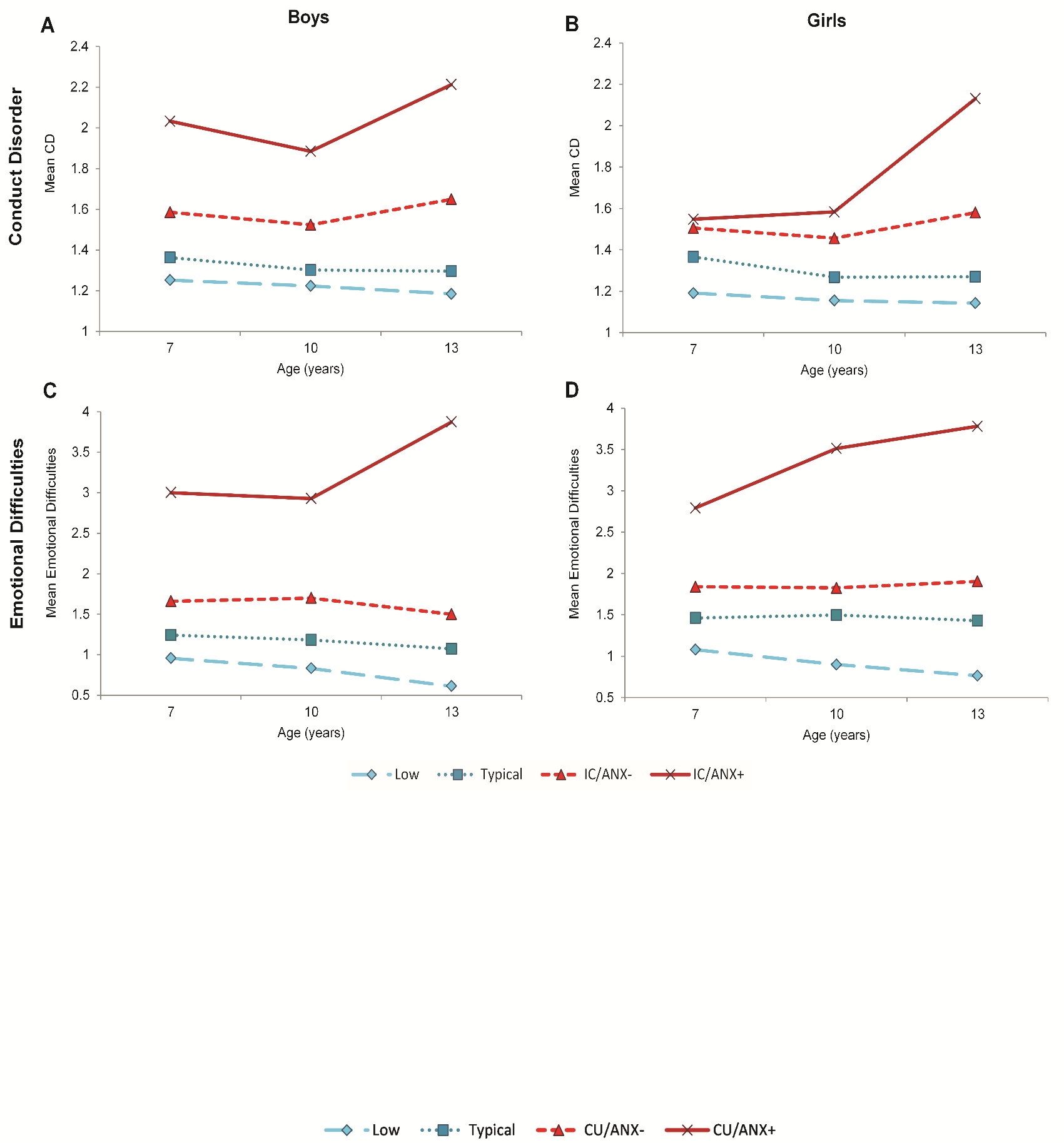


*Figure S1.* Nominally significant (i.e., *p* < .05) age × cluster × sex ANOVA interactions for conduct disorder in boys (A) and girls (B), and emotional difficulties in boys (C) and girls (D).


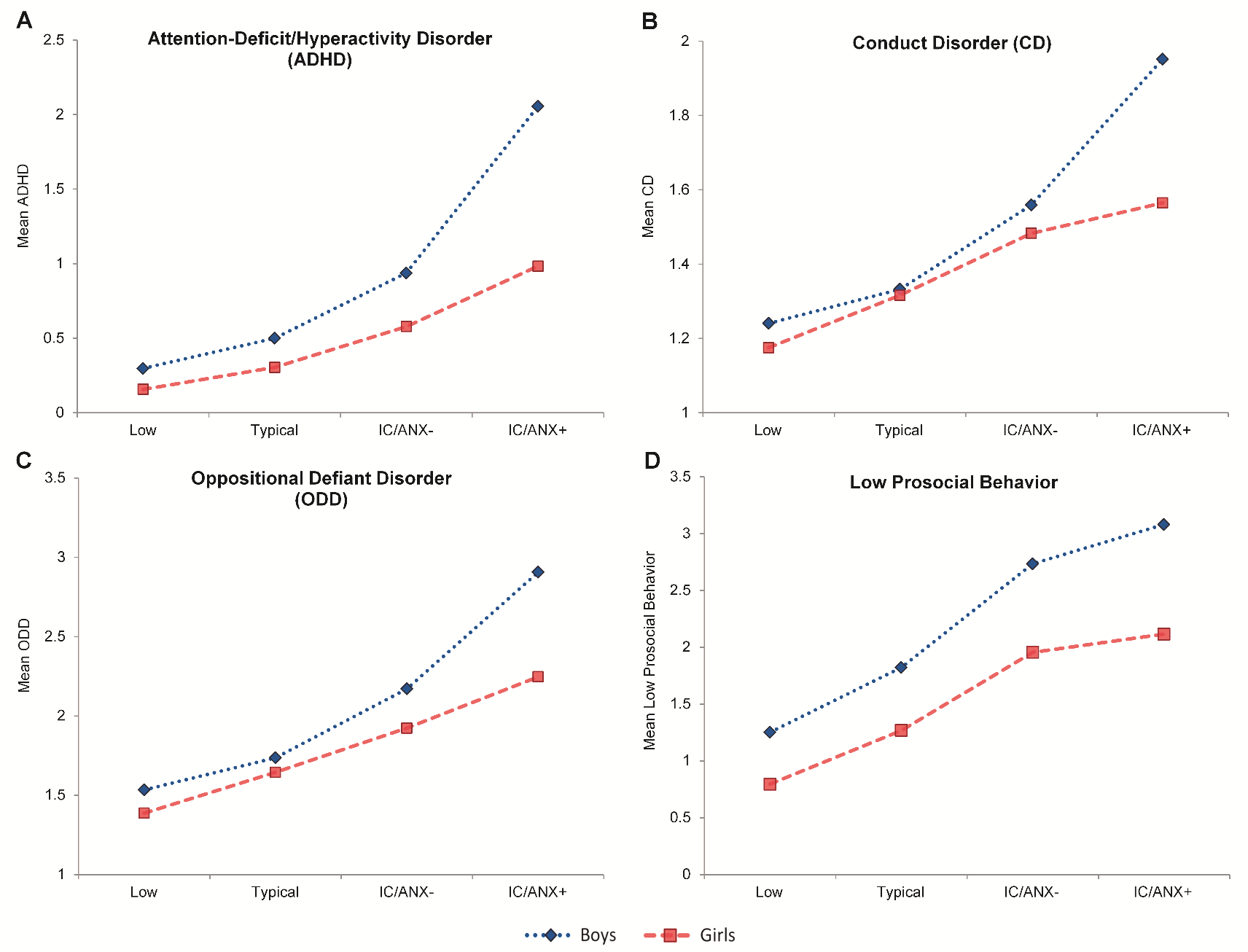


*Figure S2*. Cluster × sex ANOVA interactions between boys and girls across clusters for: (A) attention-deficit/hyperactivity disorder; (B) conduct disorder; (C) oppositional defiant disorder; and (D) low prosocial behavior.


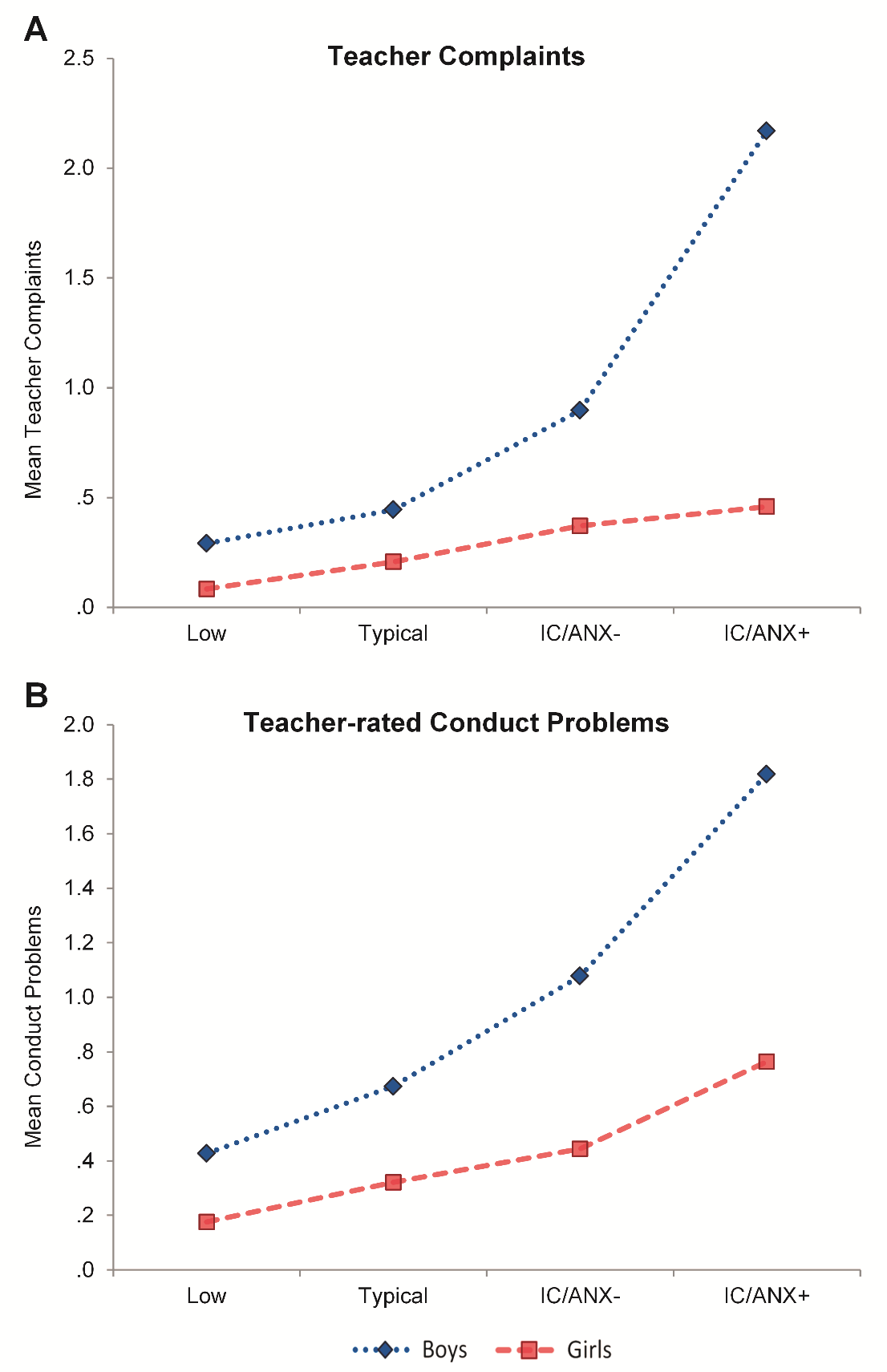


*Figure S3.* Cluster × sex ANOVA interactions between boys and girls across clusters for: (A) teacher complaints; and (B) teacher-rated conduct problems.
